# Methodology for accounting the net mitigation of China's ecological restoration projects (CANM-EP)

**DOI:** 10.1016/j.mex.2019.07.015

**Published:** 2019-07-19

**Authors:** Bojie Liu, Lu Zhang, Fei Lu, Lei Deng, Hong Zhao, Yunjian Luo, Xiuping Liu, Kerong Zhang, Xiaoke Wang, Weiwei Liu, Xueyan Wang, Yafei Yuan

**Affiliations:** a State Key Laboratory of Urban and Regional Ecology, Research Center for Eco-Environmental Sciences, Chinese Academy of Sciences, Beijing 100085, China; b China Nuclear Power Engineering Co., Ltd., Beijing 100840, China; c Joint Center for Global Change Studies, Beijing 100875, China; d State Key Laboratory of Soil Erosion and Dryland Farming on the Loess Plateau of Northwest A & F University, Yangling 712100, China; e Jinan Environmental Research Institute, Jinan 250102, China; f Department of Ecology, School of Horticulture and Plant Protection, Yangzhou University, Yangzhou 225009, China; g Center for Agricultural Resources Research, Institute of Genetics and Developmental Biology, Chinese Academy of Sciences, Shijiazhuang 050021, China; h Key Laboratory of Aquatic Botany and Watershed Ecology, Wuhan Botanical Garden, Chinese Academy of Sciences, Wuhan 430074, China; i Beijing Ecological Technology Research Institute, CIECC, Beijing 100048, China; j North China Power Engineering Co., Ltd. of China Power Engineering Consulting Group, Beijing 100011, China

**Keywords:** CANM-EP, CANM-EP, China’s ecological restoration projects, GHG budgets, net carbon sequestration

## Abstract

The real emission mitigation by the ecological restoration projects depends upon the integrated effect of all greenhouse gas (GHG) budgets rather than the carbon sequestration alone. However, a comprehensive and robust methodology for estimating the relevant GHG budgets and net mitigation of China's ecological restoration projects is still urgently to await development. Based on the methods from IPCC and statistical data of the management practices under the projects, we constructed a methodology for carbon accounting and determining net mitigation for ecological restoration projects in China (CANM-EP). GHG emissions generated from different processes and practices of the projects were included in the CANM-EP, and by this methodology, carbon sequestration, GHG balance changes induced by ecological response, on-site and off-site GHG emissions could be estimated. Therefore, the CANM-EP provides comprehensive methods to estimate the whole GHG budgets as well as the net mitigation of China's ecological restoration projects.

• The CANM-EP provides accounting methods for comprehensive processes and management practices under respective ecological restoration projects in China.
• The CANM-EP could simultaneously estimate carbon sequestration and GHG emissions of the projects.
• The CANM-EP indicates net carbon sequestration and net contribution of China's ecological restoration projects to climate change mitigation.

## Graphical abstract

**Figure:**
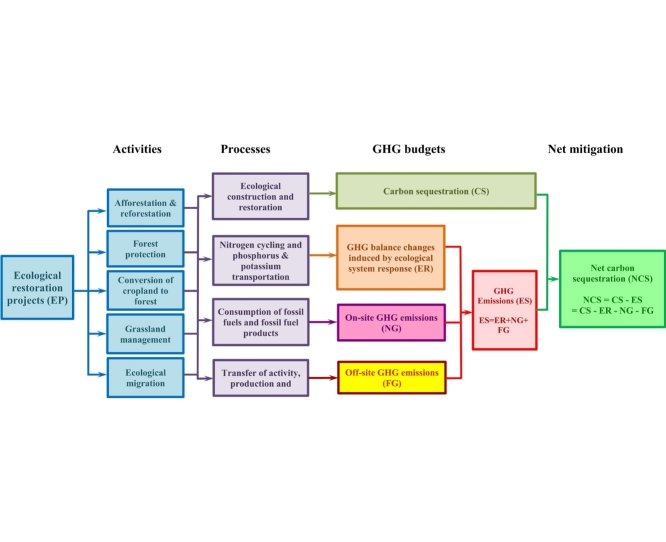

**Specifications Table**

**Table:** 

Subject Area:	Environmental Science
More specific subject area:	Climate change mitigation
Method name:	CANM-EP
Name and reference of original method:	Carbon sequestration: Chen PQ, Wang XK, Wang LM (2008) Carbon Budget and Its Sink Promotion of Terrestrial Ecosystem in China. Science Press, Beijing. IPCC (2014). Climate Change 2014: Mitigation of Climate Change. Contribution of Working Group III to the Fifth Assessment Report of the Intergovernmental Panel on Climate Change. Cambridge University Press, Cambridge, United Kingdom and New York, NY, USA. GHG emissions: IPCC (2006). IPCC Guidelines for National Greenhouse Gas Inventories. IGES, Hanagawa. IPCC (2000). Land Use, Land-Use Change and Forestry. Cambridge University Press, UK. Liu BJ, Lu F, Wang XK et al. (2016a) Greenhouse gas emissions and net carbon sequestration of the Natural Forest Protection Program in China. *Acta Ecologica Sinica*, **36**, 4266-4278. Liu BJ, Zhang L, Lu F et al. (2016b) Greenhouse gas emissions and net carbon sequestration of “Grain for Green” Program in China. *Chinese Journal of Applied Ecology*, **27**, 1693-1707.

## Method details

Since the end of the last century, China has launched a series of national ecological restoration projects with long temporal scales and large spatial scales; these projects aim to achieve general environmental improvement as well as the restoration of deteriorated ecosystems. A series of ecological management practices are employed under these ecological restoration projects to improve the regional and even the national ecosystem services [[1], [2], [3]], including afforestation and reforestation (Natural Forest Protection Project, ‘Grain-for-Green’ Project, Three-North Shelter Forest Project, Beijing-Tianjin Sand Source Control Project), forest protection (Natural Forest Protection Project), conversion of cropland to forest (‘Grain-for-Green’ Project, Beijing-Tianjin Sand Source Control Project), grassland management (Beijing-Tianjin Sand Source Control Project, Returning Grazing Land to Grassland Project) and ecological migration (Beijing-Tianjin Sand Source Control Project) [4]. Additionally, various categories of activity transfer are emerged due to the initiation of projects in the field of agriculture (‘Grain-for-Green’ Project, Beijing-Tianjin Sand Source Control Project), forestry (Natural Forest Protection Project), livestock husbandry (Beijing-Tianjin Sand Source Control Project, Returning Grazing Land to Grassland Project), energy consumption (Natural Forest Protection Project) and ecological migration (Beijing-Tianjin Sand Source Control Project) [[4], [5]].

The ecological management practices within ecological restoration projects could increase the forest and grassland area, prevent carbon loss from vegetation and soil, and subsequently enhanced carbon stocks and carbon sinks [4]. Such carbon benefits arising from ecological construction and restoration in the project region are denoted as ‘carbon sequestration’ (CS).

Nitrogen cycling and nutrient transportation induced by the projects modify the GHG budget through the ecological system response, which includes nitrous oxide (N_2_O) emissions from the application of nitrogen fertilizer in the afforestation of economic forests as well as grass planting and emissions mitigation from the reduced use of fertilizer associated with nutrient retention from decreased soil erosion. This part of carbon budget is denoted as ‘GHG balance changes induced by ecological system response’(ER) [[6], [7]].

Fossil fuels and fossil fuel products are consumed on-site with the construction and the operation of the projects, which generates ‘on-site GHG emissions’ (NG). The initiation of projects influence the transfer of activities, production and energy, including the transfer of agricultural activity, the transfer of livestock husbandry activity, the transfer of forestry activity, the ecological migration and the increased coal substitution associated with the reduced yield of firewood, which results in ‘off-site GHG emissions’ (FG). GHG emissions, including GHG balance changes induced by ecological system response, on-site GHG emissions and off-site GHG emissions negate part of increased carbon sequestration by ecological restoration projects [8]. Thus, the real, credible and verifiable emission reductions by the projects depends upon the net carbon sequestration combining the effects of all GHG emissions rather than the carbon sequestration alone [[8], [9]].

The CANM-EP method covers the major GHG emissions and leakages of afforestation, reforestation (including conversion from cropland to forest or grassland) and forest management reported around the world [10]. Therefore, it could be applied to large scale ecological restoration projects initiated elsewhere. However, the parameters of current CANM-EP methods are based on the managements and situation in China. When the CANM-EP is applied elsewhere, region-specific parameters would provide estimations with smaller uncertainties.

### Framework of CANM-EP

**Fig. 1:**
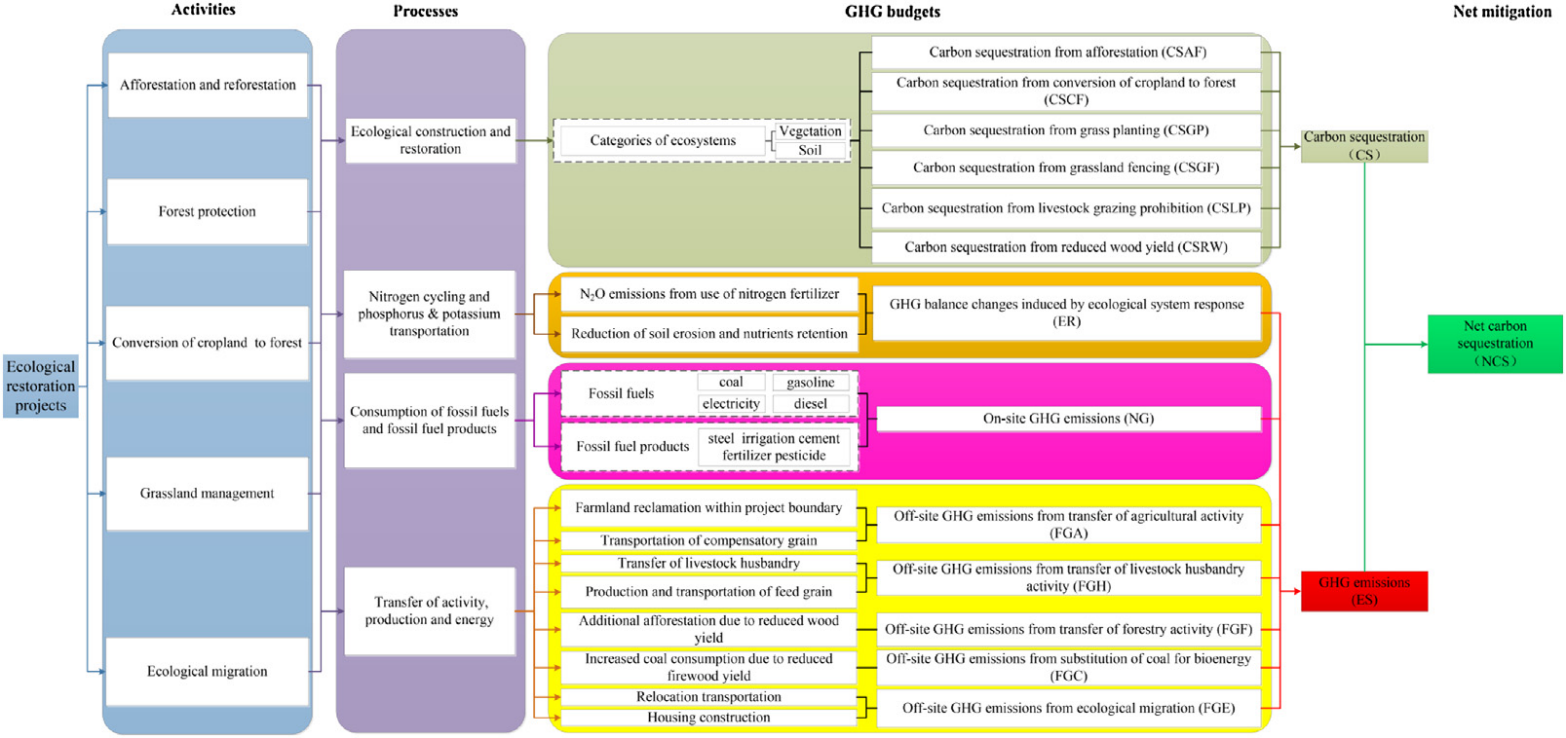
Detailed graphical framework of methodology for carbon accounting and the net mitigation of national ecological restoration projects (CANM-EP).

A methodology for carbon accounting and determining net mitigation for ecological restoration projects in China (CANM-EP) was designed, and this method was used to estimate the GHG budgets of the national ecological restoration projects as well as its net carbon mitigation. A detailed graphical framework of CANM-EP was presented as Fig.1, which shows all the possible project measures involved in ecological restoration projects and the corresponding ecosystem processes as well as the GHG budgets induced by the implementation of the project measures.

The carbon sequestration of ecological restoration projects was defined as follows:

(1) CS*_t_* = ΣCS*_kt_*

where CS*_t_* is the carbon sequestration of ecological restoration projects in the *t^th^* year (Gg C), and CS*_kt_* is the carbon sequestration from project measure *k* in the *t^th^* year (Gg C).

GHG emissions were quantified as follows:

(2) ES*_t_* = ER*_t_* + NG*_t_* + FG*_t_*

where ES*_t_* is the GHG emissions of the national ecological restoration projects in the *t^th^* year (Gg Ce), ER*_t_* is the GHG balance changes caused by the ecological system response in the *t^th^* year (Gg Ce), NG*_t_* is the on-site GHG emissions in the *t^th^* year (Gg C), FG*_t_* is the off-site GHG emissions of ecological restoration projects in the *t^th^* year (Gg C).

Based on the carbon sequestration and GHG emissions, the net carbon sequestration of the national ecological restoration projects was derived as follows:

(3) NCS*_t_* = CS*_t_* - ES*_t_*

where NCS*_t_* is the net carbon sequestration of the national ecological restoration projects in the *t^th^* year (Gg Ce).

### Carbon sequestration (CS)

#### Carbon sequestration from forest ecosystems and grassland ecosystems

Carbon sequestration from afforestation and reforestation (CSAF), conversion of cropland to forest (CSCF), grass planting (CSGP), grassland fencing (CSGF) and grazing prohibition (CSGP) was calculated as the product of the carbon sequestration rates for each project measure and the cumulative area of each project measure since the launch of the project. Thus, the carbon sequestration from each of the above project measures can be calculated via the following formula Eq. (4).

(4) CS*_kt_* = ∑ (CSR*_k_* × AS*_kt_*) × 10^-3^

where CS*_kt_* is the carbon sequestration from project measure *k* in the *t^th^* year (Gg C), CSR*_k_* is the carbon sequestration rate of project measure *k* (t C∙ha^-1^∙yr^-1^) (Table 1), and AS*_kt_* is the accumulated implementation area of project measure *k* in the *t^th^* year since the launch of the project (ha) [11].

**Table 1:** Carbon sequestration rate of project measures in different provinces.

Measures	Province	Carbon sequestration rate (t C∙ ha^-1^∙yr^-1^)	References
Afforestation and reforestation	Beijing	1.13	[12]
	Tianjin	1.13	
	Hebei	1.13	
	Shanxi	0.94	
	Inner Mongolia	1.25	
Soil retention under conversion of cropland to forest	Beijing	4.8	[1]
	Tianjin	4.8	
	Hebei	3.85	
	Shanxi	2.27	
	Inner Mongolia	0.75	
Grass planting	BTSSCP	0.54	[12]
Grassland fencing	BTSSCP	0.647	
Livestock grazing prohibition	BTSSCP	0.774	

#### Carbon sequestration from reduced wood yield (CSRW)

The reduced yield of wood is one of the project measures in ecological restoration projects that contributes to carbon sequestration via reduced logging and the corresponding loss of biomass. The carbon sequestration from a reduced wood yield was determined via the following equation Eq. (5).

(5) CSRW*_t_* = CSF_w_ × (WY*_t_* – WY_0_) × 10^-3^

where CSRW*_t_* is the carbon sequestration from the reduced yield of wood in the *t^th^* year (Gg C), CSF_w_ is the carbon sequestration factor of the wood yield reduction, 0.68 t C·m^-3^ [13], WY*_t_* is the wood yield in the *t^th^* year (m^3^) [11], and WY_0_ is the wood yield in the year before the implementation of the project (m^3^) [11].

### GHG balance changes induced by ecological system response (ER)

GHG budget changes induced by ecological system response include the carbon equivalent emissions of N_2_O released from the application of fertilizer via afforestation of economic forest and grass planting (CN*_t_*). Additionally, the emission mitigation contributed by the reduced utilization of fertilizer due to alleviated soil erosion and nutrient loss within the project region was also considered in this process (EM*_t_*). CN*_t_* and EM*_t_* were calculated via Eqs. (6) and (7), respectively.

(6) CN*_t_* = **Σ** TF*_t_* × TN% × EF_d_ × 44/28 × 298 × 10^-3^

where CN*_t_* is the carbon equivalent emissions from the N_2_O released in the *t^th^* year (Gg Ce), TF*_t_* represents the mass of the total fertilizer consumption in the *t^th^* year (t), TN% is the nitrogen content in fertilizer, which is 15% for compound fertilizer and 46.8% for urea [14], and EF_d_ is a direct emission factor of N_2_O, which is the proportion of nitrogen denitrified to N_2_O. Here, EF_d_ was derived from Zheng et al. (2014) [15] with the average values in the Northeast region, North region and South region being 0.0101, 0.00483 and 0.0119, respectively, 44/28 is the conversion coefficient between nitrogen and nitrous oxide, and 298 is the global warming potential of N_2_O relative to CO_2_ in 100 years (IPCC, 2013) [16].

(7) EM*_t_* = ∑ (NCN*_i_* - NCD*_i_*) × WER*_t_* × EF_F_*_i_* × 10^-3^

where EM*_t_* is the emission mitigation from decreased use of fertilizer in the *t^th^* year (Gg C); NCN*_i_* is the soil nutrient content i for non-degraded soil in northern China, which is 1.03, 0.32 and 2.70 g kg^-1^ for nitrogen (TN), phosphorus (P_2_O_5_) and potassium (K_2_O), respectively [17]; NCD*_i_* is the soil nutrient content i for soil degraded via wind erosion in northern China, which is 0.28-0.39, 0.13-0.17 and 2.45-2.75 g kg^-1^ for nitrogen (TN), phosphorus (P_2_O_5_) and potassium (K_2_O), respectively [17]; WER*_t_* is the reduction of wind erosion in the *t^th^* year compared with 2001 when the BTSSCP was launched (10^3^ t) [18]; and EF_F_*_i_* is the carbon emission factor of fertilizer production, which contains the nutrient *i* as the main component, and are 2.116 t C∙t N^-1^, 0.636 t C∙tP_2_O_5_^-1^ and 0.180 t C∙t k_2_O^-1^ for nitrogen fertilizer, phosphorus fertilizer and potassium fertilizer, respectively [14].

### On-site GHG emissions (NG)

#### General methods

The calculation of on-site GHG emissions induced by the utilization of fossil fuels and fossil fuel products is based on Eq. (8). The methodological approach of on-site GHG emissions is based on IPCC (2006) [19] which combines the extent of human activity with emission factors.

(8) NG*_t_* = EF*_i_* × QC*_it_* × 10^-3^

where NG*_t_* is the on-site GHG emissions in the *t^th^* year (Gg C), EF*_i_* represents the carbon emission factors of fossil fuel or fossil fuel products *i* (Table 2), and QC*_it_* is the quantity of fossil fuel or fossil fuel products *i* consumed in the *t^th^* year (t) (Eq. (9)).

**Table 2:** Carbon emission factors and related emission processes for each fossil fuel or fossil fuel product.

Category	Specific materials	Emission process	Carbon emission factor	References
Fossil fuels	Gasoline	Motorcycle patrol	0.87 t C·t^-1^	[20]
	Aviation gasoline	Aerial seeding	0.82 t C·t^-1^	[19]
	Electricity	Groundwater extraction for irrigation	0.22 kg C∙kw^-1^∙h^-1^	[8]
	Diesel	Transportation of goods	0.86 t C·t^-1^	[8]
		Site preparation for afforestation		
		Site preparation for artificial grass planting		
	Coal	Energy substitute	0.47 t C·t^-1^	[21]
Fossil fuel products	Steel	Fencing and construction of forest protection board	0.66 t C·t^-1^	[22]
	Cement	Fencing and construction of forest roads	0.19 t C·t^-1^	[23]
	Pesticide	Young forest and mature forest tending	17.28 t C·t^-1^	[24]
	2,4-D butylate herbicide	Weed control on afforestation land	2.85 t C·t^-1^	
	Trifluralin herbicide	Young forest tending	6.53 t C·t^-1^	
	Compound fertilizer	Fertilization	0.98 t C·t^-1^	[14]
	Urea	Fertilization	2.04 t C·t^-1^	

*Note*: Compound fertilizer contains the nutrients nitrogen (TN), phosphorus (P_2_O_5_), potassium (K_2_O), each of which account for 15% of the total mass. The emission factor of pesticide is the average value of the emission factors of the common forestry pesticides Fenpropathrin, Dichlorvos, Abamectin, Imidacloprid and Pyridaben.

The quantity of fossil fuel or fossil fuel products was calculated as equation Eq. (9).

(9) QC*_it_* = QU*_i_* × SP*_t_*

where QU*_i_* is the quantity of fossil fuel or fossil fuel product *i* consumed per unit of the implementation area (t∙ha^-1^) (Supplementary Table A1), and SP*_t_* is the implementation area of the project measure in the *t^th^* year (ha) [11]. The QU*_i_* values are listed in table A1 based on different fossil fuels or fossil fuel products, while the implementation areas of the respective project measures were obtained from the China Forestry Statistical Yearbook [11].

Those activities included in ecological projects can be classified into afforestation and reforestation, construction of forestry infrastructure, forest protection and grassland management measures. The methods of estimating the on-site GHG emissions induced by each activity are shown via the following equations.

#### On-site GHG emissions from afforestation and reforestation, construction of forestry infrastructure

The on-site emissions of afforestation and reforestation, and the construction of forestry infrastructure include site preparation, weed control, transportation of seedlings, irrigation, fertilization, aerial seeding, forest road construction, fence construction, and billboard construction. The emissions of fossil fuels and fossil energy products were calculated for each activity mentioned above.

##### Site preparation

The on-site GHG emissions of diesel consumption for the mechanical hoeing of land was defined as the following formula Eq. (10).

(10) NGS*_t_* = EF_D_ × QDS*_t_* × 10^−3^

where NGS*_t_* indicates the carbon emissions of the diesel consumption for site preparation in the *t^th^* year (Gg C); EF_D_ indicates the diesel carbon emission factor, 0.86 t C·t^−1^ [8]; and QDS*_t_* indicates the total amount of diesel consumption for the site preparation in the *t^th^* year (t).

##### Weed control on afforestation and reforestation land

The on-site GHG emissions of herbicide consumption for weed control on afforestation and reforestation land was estimated via the following equation Eq. (11).

(11) NGHA*_t_* = EF_HA_ × QHA*_t_* × 10^−3^

where NGHA*_t_* indicates the carbon emissions of the herbicide consumption on afforestation and reforestation land in the *t^th^* year (Gg C); EF_HA_ indicates the carbon emission factor of the commonly used herbicide 2,4-D butyl ester, 2.85 t C·t^−1^ (active ingredient) [24]; and QHA*_t_* indicates the weight of the active 2,4-D butyl ester consumed in the *t^th^* year (t).

The emissions of diesel consumption for the transportation of herbicide on afforestation and reforestation land was defined via the following equation Eq. (12).

(12) NGTHA*_t_* = EF_D_ × QDHA*_t_* × 10^−3^

where NGTHA*_t_* is the on-site emissions of diesel consumption for the transportation of herbicide in the *t^th^* year (Gg C), and QDHA*_t_* is the amount of diesel consumed for the transportation of herbicide in the *t^th^* year (t). QDHA*_t_* was calculated using the following formula Eq. (13).

(13) QDHA*_t_* = (QHA*_t_* / HAA) × UDT × RT × DD × 2 × 10^−6^

where HAA indicates the percentage of the active ingredient of 2,4-D butyl ester, which is 72% [25]; UDT is the diesel consumption per truck per hundred kilometers, which is 7 L·t^−1^·100 km^−1^ [26]; RT indicates the transportation distance, which was determined to be 100 km in this study; and DD is the diesel density (850 kg·m^−3^; a factor of 2 is applied here for the round trip).

##### Transportation of seedlings

The on-site GHG emissions of diesel consumption for seedling transport was calculated via the following formula Eq. (14).

(14) NGTS*_t_* = EF_D_ × QDTS*_t_* × 10^−3^

where NGTS*_t_* is the GHG emissions for seedling transportation in the *t^th^* year (Gg C), and QDTS*_t_* is the diesel consumption for seedling transportation in the *t^th^* year (t). The calculation for QDTS*_t_* is the same as that in Eq. (13), and the weight of the seedlings to be transported was determined using the following formula Eq. (15).

(15) QS*_t_* = (SW_1_ + SW_2_) × PD × SA*_t_* × 1/2 × 1.05 × 10^−6^

where QS*_t_* is the weight of the transported seedlings in the *t^th^* year (t); SW_1_ is the weight of the seedlings with bare roots (50 g·plant^−1^); SW_2_ is the weight of the seedlings in containers, which is 200 g·plant^−1^ (the weight factors of both types of seedlings were obtained by consulting Chengde Liyuan Garden Engineering Co., Ltd. and Beijing Sannong Agriculture Development Co., Ltd.); PD is planting density, plants·ha^−1^, which is obtained by reference to the technical regulations for afforestation and reforestation [27]; and SA*_t_* is the area of artificial afforestation and reforestation in the *t^th^* year (ha). Our study assumes that seedlings with bare roots and seedlings in containers each account for 50% of the total afforestation and reforestation area. The damage rate of seedlings during transport, and thus the replanting rate, is assumed to be 5%, so a factor of 1.05 is applied.

##### Irrigation of afforestation and reforestation

The on-site GHG emissions of afforestation and reforestation irrigation was estimated using the following equation Eq. (16).

(16) NGI*_t_* = EF_I_ × QI*_t_* × 10^−6^

where NGI*_t_* indicates the carbon emissions of afforestation and reforestation irrigation in the *t^th^* year (Gg C); EF_I_ indicates the carbon emission factor of irrigation, which is 0.02 kg C∙t^−1^ [8,28]; and QI*_t_* indicates the water consumption of the irrigation in the *t^th^* year (t).

##### Fertilization of economic afforestation and reforestation

An economically viable forest requires base fertilizer at the beginning of each year and topdressing later in the year. The most common frequency of topdressing is three times annually and usually includes stages during early flowering (flower promoting fertilizer) and late flowering (fruit promoting fertilizer). The fertilizer used for base fertilizer and topdressing is usually a nitrogen-phosphorus-potassium (NPK) compound fertilizer [2]; each nutrient in the compound fertilizer accounts for 15% of the total weight [14]. The on-site GHG emissions of fertilizer consumption for economic afforestation and reforestation was estimated via the following formula Eq. (17).

(17) NGF*_t_* = FAC × QFC*_t_* × 10^−3^ × ∑ (EF_F_*_i_*)

where NGF*_t_* is the carbon emissions of the fertilizer consumption for economic afforestation and reforestation (Gg C); FAC is the weight proportion of each nutrient (TN, P_2_O_5_, K_2_O) in the compound fertilizer, which is 15% [14]; QFC*_t_* is the total weight of the compound fertilizer consumed in the *t^th^* year (t); and EF_F_*_i_* is the carbon emission factor for the production of the *i^th^* nutrient where the nitrogen nutrition is 2.12 t C·t N^−1^, the phosphorus nutrition is 0.64 t C·t P_2_O_5_^−1^, and the potassium nutrition is 0.18 t C·t K_2_O^−1^ [14]. The on-site GHG emissions for the diesel consumption during fertilizer transportation was calculated as shown in Eqs. (12) and (13).

##### Afforestation and reforestation by aerial seeding

The on-site GHG emissions of afforestation and reforestation via aerial seeding result from the diesel consumption of the transport of seeds and aviation gasoline consumption for aerial seeding. The emissions of the diesel consumption for the seed transport were determined via the following formula Eq. (18).

(18) NGTZ*_t_* = EF_D_ × QDTZ*_t_* × 10^−3^

where NGTZ*_t_* indicates the carbon emissions of the diesel consumed during seed transport for aerial seeding in the *t^th^* year (Gg C), and QDTZ*_t_* indicates the amount of diesel consumption for the seed transportation in the *t^th^* year (t). QDTZ*_t_* can be determined using Eq. (13), in which the seed weight for aerial seeding in the *t^th^* year was calculated using the following equation Eq. (19).

(19) QZ*_t_* = UZ × SF*_t_* × 2 × 10^−3^

where QZ*_t_* indicates the seed weight for the aerial seeding in the *t^th^* year (t); UZ is the amount of aerial seeding per unit area, which is 6 kg·ha^−1^ for the northern area and 3 kg·ha^−1^ for the southern area of China [29]; and SF*_t_* is the total area for aerial seeding in the *t^th^* year (ha) [11]. The total weight is multiplied by a factor of 2 because the seed weight is twice the original weight after mechanical coating. The most commonly used airplane for aerial seeding is the Y-5 aircraft, and the corresponding on-site GHG emissions for its aviation gasoline consumption was defined as shown in the following formula Eq. (20).

(20) NGA*_t_* = EF_AG_ × QAG*_t_* × 10^−3^

where NGA*_t_* indicates the carbon emissions of the aviation gasoline consumption for the aerial seeding in the *t^th^* year (Gg C); EF_AG_ indicates the carbon emission factor of aviation gasoline, which is 0.82 t C·t^−1^ [19]; and QAG*_t_* is the amount of aviation gasoline consumed for aerial seeding in the *t^th^* year (t).

##### Construction of forests roads

The on-site GHG emissions of the building materials consumed in the construction of forest roads were calculated with the following equation Eq. (21).

(21) NGR*_t_* = EF_R_ × LR*_t_* × 10^−3^

where NGR*_t_* indicates the emissions of the building materials consumed in the construction of forest roads in the *t^th^* year (Gg C); EF_R_ is the carbon emission factor of the building materials for the construction of forest roads per kilometer, which is 86.93 t C·km^−1^ [8,23,28,30]; and LR*_t_* is the total length of the constructed forest roads in the *t^th^* year (km). LR*_t_* was determined using the following formula Eq. (22).

(22) LR*_t_* = UR × SA*_t_* × 10^−3^

where UR is the density of the forest roads, the average of which is usually 2 m·ha^-1^ in China [31]. SA*_t_* indicates the total afforestation and reforestation area in the *t^th^* year (ha) [11].

##### Construction of forest fencing

The on-site GHG emissions of the consumed building materials for fence construction were calculated with the following formula Eq. (23).

(23) NGW*_t_* = EF_F_ × LW*_t_* × 10^−3^

where NGW*_t_* indicates the carbon emissions of the consumed building materials for the fence construction in the *t^th^* year (Gg C); EF_F_ is the carbon emission factor of the fence construction per unit distance, which is 1.04 kg C·m^−1^ [8,[22], [23],^28^,[32], [33]]; and LW_t_ is the total length of the fences constructed in the *t^th^* year (km). Based on field observations, the fences are built on both sides of the forest roads to protect the vegetation on the road sides from human damage. Thus, the total length of the fencing in the present study is twice the forest road length. Thus, LW*_t_* was evaluated via the following equation Eq. (24).

(24) LW*_t_* = LR*_t_* × 2

The on-site GHG emissions for material transport was determined via the following formula Eq. (25).

(25) NGTW*_t_* = NGTS*_t_* + NGTC*_t_*

where NGTW*_t_* is the emissions for material transport for the fencing constructed in the *t^th^* year (Gg C); NGTS*_t_* indicates the emissions resulting from the steel transport in the *t^th^* year (Gg C); and NGTC*_t_* indicates the emissions resulting from the concrete transport in the *t^th^* year (Gg C). The carbon emissions of diesel consumption during the transport of steel and concrete was calculated by Eqs. (12) and (13); the weight of the transported steel was calculated via the following formula Eq. (26).

(26) QWS*_t_* = LW*_t_* × 160 × 10^−3^

where QWS*_t_* is the weight of the steel transported in the *t^th^* year (t). The weights of the 12 gage and 14 gage galvanized steel wires used for knitting the fences were 160 kg/km [33]. The total weight of the transported concrete was calculated via the following equation Eq. (27).

(27) QWC*_t_* = UVC × DWC × NWC*_t_* × 10^−3^

where QWC*_t_* is the weight of the concrete transported in the *t^th^* year (t); UVC is the volume of each cement pillar, which is 0.0288 m^3^ [33]; DWC is the density of each cement pillar, which is 2100 kg·m^−3^ [[34], [35]]; and NWC*_t_* is the number of cement pillars in the *t^th^* year. One cement pillar is needed for every 10 m section of forest fencing [33], so NWC*_t_* was determined via the following equation Eq. (28).

(28) NWC*_t_* = (LW*_t_* × 10^3^) / 10

##### Construction of billboards

The on-site GHG emissions of the materials consumed for the construction of billboards were calculated via the following equation Eq. (29).

(29) NGB*_t_* = EF_S_ × QB*_t_* × 10^−3^

where NGB*_t_* indicates the carbon emissions of the materials consumed for the construction of the billboards in the *t^th^* year (Gg C); EF_S_ is the carbon emission factor of steel production, which is 0.66 t C·t^−1^ [22]; and QB*_t_* indicates the steel weight consumed for the construction of the billboards in the *t^th^* year (t).

#### On-site GHG emissions from forest protection

Forest management and protection include pest control, tending of young forests, and patrolling the forests. The on-site emissions of the fossil fuels and fossil energy products consumed by each of these activities have been evaluated below.

##### Control of diseases and insects in forests

The commonly used forestry insecticides include Fenpropathrin, Dichlorvos, Abamectin, Imidacloprid, and Pyridaben. We assume that all these insecticides are applied for pest control, each accounting for 20% of the total. The on-site GHG emissions of insecticide consumption was determined via the following formula Eq. (30).

(30) NGP*_t_* = ∑ EF_P_*_i_* × QP*_it_* × PA*_i_* × 10^−3^

where NGP*_t_* is the carbon emissions of the insecticide consumption in the *t^th^* year (Gg C); EF_P_*_i_* is the carbon emission factor of the *i^th^* insecticide, which are 14.81, 7.80, 20.58, 20.58, and 22.64 t C·t^−1^ (active ingredient) for Fenpropathrin, Dichlorvos, Abamectin, Imidacloprid, and Pyridaben, respectively [24]; QP*_it_* is the consumption of the *i^th^* insecticide (original solution) in the *t^th^* year (t); and PA*_i_* indicates the percentage of the active ingredients of the *i^th^* insecticide, which are 20%, 80%, 1.8%, 10%, and 15% for Fenpropathrin, Dichlorvos, Abamectin, Imidacloprid, and Pyridaben, respectively [36]. The emissions of the diesel consumed for insecticide transport are determined using Eqs. (12) and (13), in which the transported insecticide amount is the sum of the amounts of all five kinds of insecticides (original solution), which is written as ∑ QP*_it_*.

##### Tending of young forests

Trifluralin is commonly used as a chemical herbicide when tending young forests [25]. The on-site GHG emissions of the Trifluralin consumed was defined via the following equation Eq. (31).

(31) NGHT*_t_* = EF_HT_ × QHT*_t_* × 10^−3^

where NGHT*_t_* indicates the carbon emissions of the Trifluralin consumed in the *t^th^* year (Gg C); EF_HT_ is the carbon emission factor for Trifluralin production, which is 6.53 t C·t^−1^ of the active ingredient [24]; and QHT*_t_* indicates the total amount of the active ingredient in Trifluralin that is used for tending young forests (t). The emissions of the diesel consumed for herbicide transport is determined using Eq. (12) and Eq. (13), in which the weight of the transported herbicide is QHT*_t_* / 0.48 and the percentage of the active ingredient in Trifluralin is 48% [25].

##### Forest patrols

The on-site GHG emissions of the fossil fuels consumed during forest patrols were determined using the following equation Eq. (32).

(32) NGMP*_t_* = EF_G_ × QG*_t_* × 10^−3^

where NGMP*_t_* indicates the carbon emissions of the gasoline consumed for motorcycle patrols in the *t^th^* year (Gg C); EF_G_ is the carbon emission factor of gasoline, which is 0.87 t C·t^−1^ [20]; and QG*_t_* indicates the weight of gasoline consumed during motorcycle patrols (t), calculated with the following formula Eq. (33).

(33) QG*_t_* = UG × UL × PF × PN*_t_* × 10^−3^

where UG is the weight of gasoline consumed per kilometer per motorcycle (0.0145 kg·km^−1^), UL is the patrol distance for each motorcycle per each trip (100 km/ motorcycle / patrol time) [37], PF is the patrol time for each motorcycle per each year (300 times·motorcyle^−1^·yr^−1^) [37], and PN*_t_* indicates the number of motorcycles required for each forest patrol in the *t^th^* year.

(34) PN*_t_* = (SP*_t_* / 380) × 1/4

where SP*_t_* is the area of forest requiring management and protection (ha) [11]. Each forest ranger is responsible for 380 ha [38]. In the present study, we assume that 1/4 of the rangers have motorcycles; thus, the number of motorcycles is the number of rangers multiplied by a factor of 1/4.

#### On-site GHG emissions from grassland management

Grassland management includes grass planting, grassland fencing, and grazing prohibition. Grass planting comprises site preparation, grass seed transportation, irrigation, and fertilization. The carbon emissions resulting from the diesel consumption for site preparation can be calculated using Eq. (10). Here, we introduce the on-site GHG emissions calculations for grass seed transportation, grassland irrigation, and grassland fertilization. Grassland fencing includes fence construction and the transport of the corresponding goods; grazing prohibition involves the construction of sheds for feeding.

##### Grass planting

(1) Transport of grass seed

The carbon emissions produced from the diesel consumption required for grass seed transport is determined using Eqs. (12) and (13), in which the weight of transported grass seed was calculated via the following equation Eq. (35).

(35) QGS*_t_* = UGS × SRG*_t_* × 2 × 10^−3^

where QGS*_t_* is the weight of the grass seed transported in the *t^th^* year (t); UGS is the seed amount per unit area, which is 15 kg·ha^−1^ [33]; and SRG*_t_* indicates the area of artificial grass planting in the *t^th^* year (ha) [11]. The seed weight is twice the original weight after mechanical coating, so the total weight is the original weight multiplied by a factor of 2.

(2) Grassland irrigation

The on-site GHG emissions of the water consumed for grassland irrigation was defined via the following equation Eq. (36).

(36) NGIG*_t_* = EF_I_ × QIG*_t_* × 10^−6^

where NGIG*_t_* is the carbon emissions of the irrigation in the *t^th^* year (Gg C); EF_I_ indicates the carbon emission factor of irrigation, which is 0.02 kg C∙t^−1^ [8,28]; and QIG*_t_* is the irrigation volume in the *t^th^* year (t).

(37) QIG*_t_* = UIG × ASRG*_t_*

where UIG indicates the irrigation quota in each year, which is 4,000 t·ha^−1^·yr^−1^ [33]; and ASRG*_t_* is the cumulative area of artificial grass planting in the *t^th^* year (ha) [11].

(3) Grassland fertilization

Grassland fertilization comprises the application of base fertilizer, seed fertilizer, and topdressing. Base fertilizer is the organic fertilizer applied to the soil before the grass planting, which provides the nutrients to the plants throughout the growing season. Seed fertilizer is mainly inorganic fertilizer and supplies nutrition during the seedling period. Quick acting inorganic fertilizer is the major component of topdressing; this fertilizer is applied to replenish certain nutrients during a particular stage of plant growth [33]. The organic fertilizer is mainly fecaluria manure, green manure, and farmyard manure and thus does not impact the on-site GHG emissions of this aspect of the forestry production process [39]. Therefore, the on-site GHG emissions of the production and transportation of the seed fertilizer and topdressing are evaluated in the present study. Nitrogen-phosphorus-potassium (NPK) compound fertilizer is commonly used as seed fertilizer, and inorganic urea usually serves as the top dressing. The carbon emissions of the consumption of seed fertilizer was calculated via the following equation Eq. (38).

(38) NGGB*_t_* = ∑ (EF_F_*_i_*) × FAC × QFCG*_t_* × 10^−3^

where NGGB*_t_* indicates the carbon emissions of the NPK compound fertilizer consumed as seed fertilizer in the *t^th^* year (Gg C). Additionally, EF_F_*_i_* is the carbon emission factor for the production of the *i^th^* nutrient; specifically, nitrogen nutrition is 2.12 t C·t N^−1^, phosphorous nutrition is 0.64 t C·t P_2_O_5_^−1^ and potassium nutrition is 0.18 t C·t K_2_O^−1^ [14]. FAC indicates the percentage of each nutrient in the compound fertilizer, which is 15% of the total mass [14], and QFCG*_t_* is the weight of the compound fertilizer consumed as seed fertilizer in the *t^th^* year (t).

(39) QFCG*_t_* = UFGB × SRG*_t_* × 10^−3^

where UFGB is the amount of seed fertilizer applied per unit area, which is 75 kg·ha^−1^ [33,40]. The emissions of the diesel consumed for the compound fertilizer transport is determined using Eqs. (12) and (13), and QFCG*_t_* is the weight of the transported compound fertilizer. The on-site GHG emissions of the topdressing consumption were calculated according to the following equation Eq. (40).

(40) NGGT*_t_* = EF_FN_ × FAN × QFNG*_t_* × 10^−3^

where NGGT*_t_* indicates the carbon emissions of the urea application in the *t^th^* year (Gg C); EF_FN_ is the carbon emission factor of the nitrogen in the urea, which is 2.04 t C·t^−1^ [14]; FAN is the percentage of the nitrogen in the urea (46.8%) [14]; and QFNG*_t_* indicates the weight of the urea consumed as topdressing (t).

(41) QFNG*_t_* = UFGT × ASRG*_t_* × 3 × 10^−3^

where UFGT is the amount of topdressing applied, which is 110 kg·ha^−1^ [33,41]; and the frequency of the topdressing application is three times per year [[33], [34], [35], [36], [37], [38], [39], [40], [41]]. The emissions of the diesel consumed for urea transport are determined by Eq. (12) and (13), and QFNG*_t_* is the weight of the urea to be transported.

##### Grassland fencing

The on-site GHG emissions of the building materials used for fence construction were defined as follows Eq. (42).

(42) NGWG*_t_* = EF_F_ × LWG*_t_* × 10^−3^

where NGWG*_t_* indicates the carbon emissions of the building materials used for fence construction in the *t^th^* year (Gg C); EF_F_ is the carbon emission factor for the fences constructed per unit distance, which is 1.04 kg C·m^−1^ [8,[22], [23],^28^,[32], [33]]; and LWG*_t_* indicates the length of the fencing constructed in the *t^th^* year (km).

(43) LWG*_t_* = (SFG*_t_* / 50) × 2830 × 10^−3^

where SFG*_t_* indicates the area of enclosed grassland in the *t^th^* year [11], wherein the unit area of enclosed grassland is 50 ha, and the length of the corresponding fences is 2,830 m [33]. The emissions of the diesel consumed during the transport of the steel and concrete was determined using Eqs. (12) and (13), in which the weight for the steel and concrete was determined by Eqs. (26) and (27).

##### Grazing prohibition

The on-site GHG emissions of the building materials for shed construction were derived as follows Eq. (44).

(44) NGSN*_t_* = EF_SN_ × SSN*_t_* × 10^−6^

where NGSN*_t_* indicates the carbon emissions of the building materials for shed construction. The pens for cows and sheep are usually cuboids with a length of 60 m, a width of 10 m, average heights of 4.5 m (cow pen) or 2.5–3 m (sheep pen), and a wall thickness of 0.24 m. Therefore, the volume of each shed is calculated to be 118 m^3^. The construction of each cubic meter of wall requires 522 standard bricks, 64.6 kg of cement, and 9.68 kg of water. Based on the carbon emission factors of the above building materials, the carbon emission factor of shed construction per unit area (EF_SN_) is calculated to be 15.31 kg C·m^−2^ [8,23,28]. SSN*_t_* is the construction area of a shed in the *t^th^* year (m^2^) [11,42].

### Off-site GHG emissions (FG)

#### Off-site GHG emissions from the transfer of agricultural activity (FGA)

Off-site GHG emissions from the transfer of agricultural activities (FGA) are generated from the transport of compensatory grain and farmland reclamation due to conversion of farmland to forest or grassland within the project region. The off-site carbon emissions from the transport of grain and farmland reclamation was calculated via Eqs. (45) and (52), respectively.

##### Transport of compensatory grain for the conversion of cropland to forest

The provision of compensatory grain to farmers by the government is one of the main aspects of the conversion of cropland to forest. Compensatory grain was supplied directly to farmers from 2001 to 2003 and has been converted to subsidies since 2004 due to an adjustment of the compensatory grain policy [37]. Additional GHG emissions are generated via fossil fuel combustion when farmers transport the compensatory grain supplied by the government directly or bought using subsidies.

(45) FGTG*_t_* = EF_D_ × QDTG*_t_* × 10^-3^

where FGTG*_t_* is the off-site GHG emissions from diesel consumed in the transport of compensatory grain in the *t^th^* year (Gg C); EF_D_ is the carbon emission factor of diesel, 0.86 t C∙t^-1^ [8]; and QDTG*_t_* is the mass of the diesel fuel consumed for grain transport in the *t^th^* year (t). QDTG*_t_* was calculated via Eq. (46).

(46) QDTG*_t_* = 2 × UDT × DD× (TGI*_t_* × RGI + TGE*_t_* × RGE) × 10^-6^

where UDT is the diesel fuel consumption rate when a truck travels 100 km with one ton of cargo, specifically, 7 L∙t^-1^∙100 km^-1^ [26]; DD is the density of diesel, i.e., 850 kg∙m^-3^; TGI_t_ is mass of grain transported within a county in the *t^th^* year (t); RGI is the average distance over which grain is transported within a county (km); TGE*_t_* is the mass of the inter-county grain transport in the *t^th^* year (t); and RGE is the average distance of the inter-county grain transport (km).

The annual mass of transported grain was estimated on the basis of the grain supplied by the government directly and the grain bought using subsidies via the following equation Eq. (47).

(47) TG*_t_* = GG*_t_* + (SG*_t_* / α) ∙ β ∙ 10

where TG*_t_* is the mass of the grain transported in the *t^th^* year (t); GG*_t_* is the mass of the compensatory grain supplied directly by the government in the *t^th^* year (t); SG*_t_* is the subsidy for the compensatory grain supplied by the government in the *t^th^* year (10^4^ RMB); α is the price for 1 kg of grain, i.e., 1.4 RMB∙kg^-1^ [37]; and β is the modified coefficient of the grain mass when the labor migration is considered, i.e., 0.7. Based on an enquiry from the “Grain for Green” office of the State Forestry Administration, the grain reserved within each county could satisfy 80% of the compensatory grain while the other 20% comes from neighboring counties. Therefore, the masses of the grain transported within a county and between counties are shown as follows.

(48) TGI*_t_* = TG*_t_*

(49) TGE*_t_* = 0.2TG*_t_*

where TGI*_t_* is the mass of the grain transported within a county in the *t^th^* year (t), and TGE*_t_* is the mass of the grain transported between counties in the *t^th^* year (t).

We assume that the shape of each project county was square and that the average distance of grain transport within each county was a quarter of the square's diagonal Eq. (50).

(50) $$\text{R}\text{G}\text{I}=\frac{\sqrt{2\text{C}\text{A}}}{4}$$

where RGI is the average distance of grain transport within a county (km), and CA is the area of each project county (km^2^). Similarly, we assume that the shape of each project province was also a square and that the average distance of the grain transport between counties was calculated via the following equation Eq. (51).

(51) $$\text{R}\text{G}\text{E}=\sqrt{\frac{\text{P}\text{A}}{\text{N}}}$$

where RGE is the average distance over which the grain was transported between counties (km), PA is the area of each project province (km^2^), and N is number of project counties per project province.

In the frame of conversion of cropland to forest, the amount of compensation grain was set in accordance with the yield of the hilly or poor croplands before their conversion [43]. Using this methodology, the amount of compensation grain is assumed to be almost the same as the yield of the cropland before conversion, and subsequently, it could be considered as the grain production transferred from the project area to other agricultural regions together with the GHG emissions during the production processes. The GHG emissions due to fertilizer and pesticide applications, machinery operations, and irrigation move from the conversion area to other agricultural regions; therefore, the changes in the total GHG emissions due to grain production at the national or regional scales are not considered [5].

##### Farmland reclamation

In addition to the transport of compensatory grain, off-site GHG emissions were also generated by the reclamation of farmland. Due to a decreased farmland area, the production of grain declined in the early period of the project, during which the conversion of cropland to forest was implemented [44], resulting in increased grain prices and the expansion of farmland [45]. Accordingly, carbon emissions increased due to increased vegetation and soil carbon loss when forestland, shrubs and grassland were converted into farmland. In this study, we hypothesized that the reclamation of farmland within each project county resulted from the implementation of conversion of cropland to forest. Off-site GHG emissions from the reclamation of farmland were calculated according to the following equation Eq. (52).

(52) FGRF*_t_* = FGV*_t_* + FGS*_t_*

where FGRF*_t_* is the carbon emissions due to the reclamation of farmland from forestland, shrubs and grassland in the *t^th^* year (Gg C), and FGV*_t_* and FGS*_t_* are the carbon emissions from the loss of vegetation and soil, respectively, in the year *t* (Gg C). FGV*_t_* and FGS*_t_* were calculated based on Eqs. (53) and (54), respectively.

(53) FGV*_t_* = (ΔDVF × SRF*_t_* + ΔDVS × SRS*_t_* + ΔDVG × SRG*_t_*) × 10^-3^

where ΔDVF, ΔDVS and ΔDVG are the carbon density losses due to vegetation for forestland, shrubs and grassland, respectively (t C∙ha^-1^). SRF*_t_*, SRS*_t_* and SRG*_t_* are the areas of farmland reclamation from forestland, shrubs and grassland, respectively, in the *t^th^* year (ha). The carbon density losses due to vegetation for forestland, shrubs and grassland in the various regions of China are listed in Table 3. SRF_t,_ SRS_t_ and SRG_t_ were calculated based on land use data coming from the ChinaCover databases produced by the Chinese Academy of Sciences. Such databases take domestic satellite HJ as the main data source, using resolutions of 30 m and assisted by Landsat TM data, and the data processing was automatic due to the use of a supercomputing platform. Object-oriented technology was adopted for the classification of remote sensing imagery based on system characters, and three first-level classifications were adopted from related studies to measure the farmland conversion area. Based on superposition and the tests of 31,675 random sampling points, the resolutions of the classifications for forestland, shrubs, grassland and farmland were 96%, 95%, 93% and 94%, respectively [[46], [47]]. We obtained the land use data within the counties for the conversion of cropland to forest.

(54) FGS*_t_* = (ΔDSF × SRF*_t_* + ΔDSS × SRS*_t_* + ΔDSG × SRG*_t_*) × 10^-3^

where ΔDSF, ΔDSS and ΔDSG are the differences of the carbon densities for the top soil (0-20 cm) between forestland, shrubs, grassland and farmland, respectively (t C∙ha^-1^) (Table 3).

**Table 3:** Loss of carbon density for vegetation and soils when forestland, shrubs and grassland were converted to farmland in the respective regions of China.

Region	Loss of carbon density for vegetation (t C·ha^-1^)			Loss of carbon density for soils (t C·ha^-1^)			References
	Forestland	Shrubs	Grassland	Forestland	Shrubs	Grassland	
Northwest	45.05	6.53	2.73	76.77	15.50	0.53	[[48], [49], [50], [51], [52], [53], [54], [55], [56], [57], [58]]
Southwest	52.87	13.47	3.98	41.13	0	0	
Northeast	43.83	6.24	4.95	49.77	0	0	
North	24.34	6.23	3.77	27.95	4.06	10.04	
Central south and east	25.79	12.51	3.61	34.95	0	4.92	

#### Off-site GHG emissions from the transfer of livestock husbandry activity (FGH)

Prohibiting grazing and grassland fencing are the two main measures of grassland management in the BTSSCP and are meant to alleviate land desertification and improve the structures of animal husbandry in the project region [42]. GHG emissions were generated via additional production and activity transfer.

##### Off-site GHG emissions from the production and transport of compensatory feed grain

Distinct from the off-site GHG emissions from the grain compensation due to land conversions from cropland to forest, under the management of grazing prohibitions, foraging supply decreases and additional feed grain (usually aged grain) is required to raise livestock, leading to additional GHG emissions during the production of this grain.

Compensatory feed grain was supplied to regions implementing grazing prohibition in the BTSSCP region for five years. The standard for the distribution of the compensatory feed grain in Inner Mongolia was 82.5 kg grain·ha^-1^·yr^-1^, while in Beijing, Tianjin, Hebei and Shanxi, the standard was 40.5 kg grain·ha^-1^·yr^-1^ [59]. Therefore, off-site carbon emissions were generated from the production and transport of the compensatory feed grain.

(55) FGPT*_t_* = FGFP*_t_* + FGFT*_t_*

where FGFP*_t_* is the carbon emissions generated by the production of compensatory feed grain in the *t^th^* year (Gg C), and FGFT*_t_* is the carbon emissions generated from the transport of compensatory feed grain in the *t^th^* year (Gg C). FGFP*_t_* was calculated as equation Eq. (56).

(56) FGFP*_t_* = QFG*_t_* × (FC × EF_FC_ + FS × EF_FS_ + FW × EF_FW_) × 10^-3^

where QFG*_t_* is the mass of the compensatory feed grain in the *t^th^* year (t) [11], and FC, FS and FW are proportions of corn, soybean and wheat in compensatory feed grain, respectively. According to the ingredients of the feed grain used for lambs in China, corn, soybean and wheat account for 50%, 10% and 40% of the grain, respectively. EF_FC_, EF_FS_ and EF_FW_ are the carbon emission factors for producing corn, soybean and wheat, respectively. Cheng et al. (2015) [60] reported that the carbon footprints of corn, soybean and wheat in China were 0.12, 0.10 and 0.14 kg C/kg product, respectively. The quantity of diesel fuel consumed in the transport of the compensatory feed grain was calculated via Eq. (57).

(57) QDFT*_t_* = 2 × QFG*_t_* × RF × UDT × DD × 10^-6^

where QDFT*_t_* is the mass of diesel fuel consumed during the transport of the compensatory feed grain for the year *t* (t); RF is the distance that the compensatory feed grain is transported (km); UDT is the diesel consumption per truck per hundred kilometers, which is 7 L·t^−1^·100 km^−1^ [26]; and DD is the diesel density (850 kg·m^−3^); a factor of 2 is applied here for the round trip). Thus, the carbon emissions from the transport of the compensatory feed grain were defined via the following equation (Eq. 58).

(58) FGFT*_t_* = EF_D_ × QDFT*_t_* × 10^-3^

where FGFT*_t_* is the carbon emissions generated during the transport of compensatory feed grain during the *t^th^* year (Gg C), and EF_D_ indicates the diesel carbon emission factor, i.e., 0.86 t C·t^−1^ [8].

##### Off-site GHG emissions from extended off-site overgrazing

(1) Background of extended off-site overgrazing in Inner Mongolia

In addition to the production and transport of compensatory feed grain, off-site GHG emissions were also generated from the overgrazing of grasslands that resulted from the transfer of livestock husbandry activities from within-project counties to the counties outside of the project area. During the project period of 2001-2010, the proportions of bovine and caprine stocks as stocking sheep unit (SU) within the project regions of Beijing, Tianjin, Hebei and Shanxi to the total stocking SU in the corresponding provinces fluctuated with no obvious regularity. Thus, it is difficult to obtain the relationship between the implementation of the BTSSCP and the dynamics of the livestock amounts, which is regarded as a key element of the GHG leakage. However, the proportions in Inner Mongolia declined during the period of the BTSSCP. At the same time, the proportion of stocking SU in Inner Mongolia to that of the total SU in China increased during the project period [44]. Thus, we assume that the animal husbandry industry of the whole Inner Mongolia was not hindered by the BTSSCP.

**Fig. 2:**
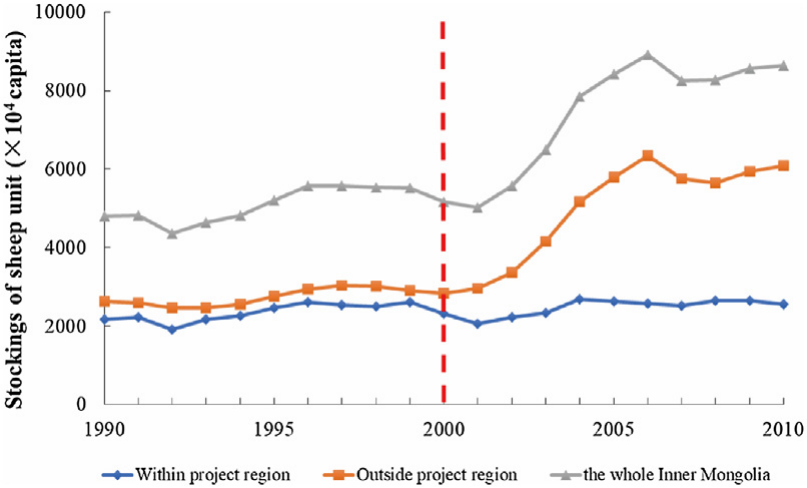
Variances of stocking SU within the project region, outside of the project region and over the whole Inner Mongolia from 1990 to 2010.

Furthermore, we compared the variance of the stocking SU in the whole Inner Mongolia, counties within the project region and counties outside the BTSSCP during 1990-2010 (Fig.2). We found that before the implementation of BTSSCP (1990-2000), the trends of the variances of the stocking SU were similar within the project region, outside the project region and over the whole of Inner Mongolia. However, since 2001, when the BTSSCP started, the variance of the stocking SU within the project region did not change obviously, while the stocking SU outside the project region and for the whole of Inner Mongolia increased significantly (Fig.2). Thus, we assume that the portion of the stocking SU which would otherwise have been reared within the project region of Inner Mongolia was transferred to regions outside of the project region.

(2) Calculation of off-site GHG emissions from extended off-site overgrazing in Inner Mongolia

Based on the analysis mentioned above and the fact that the area of grassland fencing in Inner Mongolia accounts for over 70% of the total area of grassland fencing in the BTSSCP, the transfer of livestock husbandry mainly occurred within Inner Mongolia. We assume that this transfer was confined to within Inner Mongolia, according to China’s relative regulations and systems. The transfer of livestock could exert additional grazing pressure on grasslands, which would induce a decline in the soil organic carbon due to overgrazing [51,61].

We obtained a database from the Agricultural Information Institution, Chinese Academy of Agricultural Sciences. This database supplied stocking data of bovine and caprine for 75 counties within the BTSSCP at annual intervals from 2000 to 2010. The stocking of bovine and caprine in Inner Mongolia was calculated as SU using the following equation Eq. (59).

(59) NS = 5 × NB + NC

where NS is the stocking number of SU in Inner Mongolia (capita), NB is the stocking number of bovine in Inner Mongolia (capita), and NC is the stocking number of caprine in Inner Mongolia (capita). The stock-carrying capacity per unit grassland outside of the project region of the BTSSCP was calculated, and the grazing intensity was evaluated based on the different grassland types and their areas. A baseline scenario was designed for the year 2000 before the implementation of the BTSSCP. We assumed that under the baseline scenario, the proportion of stocking SU outside of the project region to the total stocking SU in Inner Mongolia in the year t was the same as the proportion in the year 2000 (Eq. (60)).

(60) NSO_b0_ / NS_b0_ = NSO_b_*_t_* / NS_b_*_t_*

where NSO_b0_ is the stocking number of SU outside of the project region in the year 2000 in Inner Mongolia (capita) [44,62], NS_b0_ is the stocking number of SU in the year 2000 in the whole of Inner Mongolia (capita) [44], NSO_b_*_t_* is the stocking number of SU outside of the project region under the baseline scenario in the year *t* in Inner Mongolia (capita), and NS_b_*_t_* is the stocking number of SU under the baseline scenario in the year *t* over the whole of Inner Mongolia (capita) [44]. Given that the husbandry industry across the whole of Inner Mongolia was not affected by the BTSSCP, the stocking number of SU in Inner Mongolia under the baseline scenario was the same as the stocking number under the project scenario. Thus, the stocking number of SU outside of the project region under the baseline scenario in the *t^th^* year in Inner Mongolia was determined as

(61) NSO_b_*_t_* = (NSO_b0_ / NS_b0_) × NS_b_*_t_*

Under the project scenario, the stocking number of SU outside of the project region in the *t^th^* year in Inner Mongolia was obtained by Eq. (62).

(62) NSO_p_*_t_* = NS_p_*_t_* – NSI_p_*_t_*

where NSO_p_*_t_* is the stocking number of SU outside of the project region under the project scenario in the *t^th^* year in Inner Mongolia (capita), NS_p_*_t_* is the stocking number of SU under the project scenario in the *t^th^* year in Inner Mongolia (capita) [44], and NSI_p_*_t_* is the stocking number of SU in the project region under the project scenario in the *t^th^* year in Inner Mongolia (capita) [62]. Therefore, the transfer of stocking SU from within the project region to outside of the project region in Inner Mongolia was defined as

(63) NT*_t_* = NSO_p_*_t_* – NSO_b_*_t_*

where NT*_t_* is the transfer of stocking SU from within the project region to outside of the project region in the year *t* in Inner Mongolia (capita). We assumed that the proportion of SU transferred from within the project region to each county outside of the project region to the total SU transferred from within the project region to counties outside of the project region is the same as the proportion of increased SU in each county outside of the project region to the total increased SU in those counties outside of the project region (Eq. 64).

(64) NT*_nt_* / NT*_t_* = (NSO*_nt_* – NSO*_n(t-1)_*) / (NSO*_t_* - NSO*_t-1_*)

where NT*_nt_* is the transfer of SU from within the project region to county n outside the project region in the *t^th^* year (capita); NSO*_nt_* and NSO*_n(t-1)_* are the stocking numbers of SU in county *n* outside the BTSSCP in the years *t* and *t-1*, respectively (capita); and NSO*_t_* and NSO*_t-1_* are the total stocking numbers of SU in counties outside the BTSSCP in the years *t* and *t-1*, respectively (capita). Therefore, the transfer of SU from within the project region to county n outside the BTSSCP in the *t^th^* year was calculated as

(65) NT*_nt_* = (NSO*_nt_* - NSO*_n(t-1)_*) / (NSO*_t_* - NSO*_t-1_*) × NT*_t_*

Moderate stock-carrying capacities per unit area of typical grasslands and desert grasslands are 4.5 capita·ha^-1^ and 1.82 capita·ha^-1^, respectively [[63], [64]], and the theoretical moderate stock-carrying capacity for each county outside the BTSSCP in Inner Mongolia was determined to be

(66) MSO*_n_* = STO*_n_* × 4.5 + SDO*_n_* × 1.82

where MSO*_n_* is the moderate stock-carrying capacity in county *n* outside the BTSSCP in Inner Mongolia (capita), STO*_n_* is the area of typical grasslands for county *n* outside the BTSSCP (ha), and SDO*_n_* is the area of the desert grasslands for county n outside the BTSSCP (ha). The areas of the grasslands in each county were adopted from the ChinaCover datasets produced by the Chinese Academy of Sciences, and the areas for the different categories of grasslands were measured via remote sensing image classification [47]. The areas of grasslands and the corresponding theoretical moderate stock-carrying capacities for each county outside the BTSSCP in the year 2000 were set as the baseline scenario. According to the theoretical moderate stock-carrying capacity in 2000 and the actual stocking numbers of SU in each year, the extents of overgrazing in each county outside the BTSSCP were defined as

(67) EO*_nt_* = NSO*_nt_* / MSO*_n_*_0_

where EO*_nt_* is the degree of overgrazing in county n outside the BTSSCP in the *t^th^* year. According to Qi (2005) [63], the intervals for moderate grazing, over grazing and severe over grazing are EO < 1, 1 < EO < 3 and EO > 3, respectively. NSO*_nt_* is the actual stocking number of SU in county *n* outside the BTSSCP in the *t^th^* year (capita) [44,62], and MSO*_n_*_0_ is the theoretical moderate stock-carrying capacity for county *n* outside the BTSSCP in 2000 (capita). If the extent of over grazing in county n changes from moderate grazing to over grazing or severe over grazing for two consecutive years, we assume that the increased stockings were due to the transfer of livestock husbandry activity, inducing the deterioration of grasslands. The off-site GHG emissions generated by county *n* outside the project region, which was converted from “moderate grazing” in the previous year to “over grazing” or “extreme over grazing” in the following year, was calculated via Eq. (68).

(68) FGOG*_nt_* = (STO*_nt_* × 0.774 + SDO*_nt_* × 0.379) × 10^-3^

where FGOG*_nt_* is the off-site carbon emissions from over grazing due to the transfer of livestock husbandry in county *n* outside the project region in the *t^th^* year (Gg C), STO*_nt_* is the area of typical grasslands in county *n* outside the project region of Inner Mongolia in the *t^th^* year (ha), and SDO*_nt_* is the area of desert grasslands in county *n* outside the project region of Inner Mongolia in the *t^th^* year (ha); the carbon emission rates of increasing grazing pressure on typical grasslands and desert grasslands are 0.774 t C·ha^-1^·yr^-1^ and 0.379 t C·ha^-1^·yr^-1^, respectively [12]. According to Hopkins et al. (2007) [65], methane emissions were not involved in the transfer of livestock on grassland ecosystems under the BTSSCP. The off-site GHG emissions from overgrazing in the outer project region of Inner Mongolia are the sum of the carbon emissions from those counties considered Eq. (69).

(69) FGOG*_t_* = ∑FGOG*_nt_*

#### Off-site GHG emissions from the transfer of forestry activities (FGF)

Along with the economic development of China, the supply and demand of timber did not decrease gradually [11]. In some of the key national ecological restoration projects, such as the Natural Forest Resource Protection, the commercial harvest of timber was forbidden within the project region. Therefore, this part of the reduced timber supply due to ecological restoration projects needs to be produced off-site.

The off-site GHG emissions generated via the consumption of fossil fuels and fossil fuel products during the process of afforestation and reforestation outside of the project boundaries were defined via the following formula Eq. (70).

(70) FGF*_t_* = EF_a_ × SF*_t_* ×10^-3^

where FGF*_t_* is carbon emissions from transfer of forestry activities in the *t^th^* year (Gg C), EF_a_ is GHG emissions from unit area of afforestation and reforestation for timber forest, which was cited from Liu et al. (2016) [13] (t C·ha^-1^), and SF*_t_* is additional area of afforestation and reforestation for timber forests (ha).

Additional areas of afforestation and reforestation for timber forests due to the reduced supply of logs within the project region were calculated via Eqs. (71), (72), (73).

(71) WYF*_t_* = (WY*_t_* - WY_0_) × PF*_t_*

where WYF*_t_* is the reduced yield of firewood in the *t^th^* year (m^3^), WY*_t_* is the yield of wood in the *t^th^* year (m^3^) [11], WY_0_ is the yield of wood in the year before the wood yield reduction measures were launched [11], and PF*_t_* is the proportion of firewood yield to the total wood yield in the *t^th^* year (%). Due to a shortage of data on firewood yield reduction, we assume that the proportion of firewood yield reduction to total wood yield reduction in the *t^th^* year was the same as the percentage of firewood yield of the total wood yield in the *t^th^* year.

(72) WYL*_t_* = (WY*_t_* - WY_0_) - WYF*_t_*

where WYL*_t_* is the reduced yield of logs in the *t^th^* year (m^3^). The area of the additional afforestation and reforestation for timber forests outside the project boundary was calculated via Eq. (73).

(73) SF*_t_* = (WYL*_t_* / 0.59) / UV

where SF*_t_* is the area of afforestation and reforestation for timber forests in the *t^th^* year (ha), 0.59 is the recovery of commercial timber [49], and UV is the forest volume per unit area as cited from the 7^th^ National Forest Inventory in China (m^3^ ha^-1^).

#### Off-site GHG emissions from the substitution of coal for bioenergy (FGC)

In some of the key national ecological restoration projects (e.g., Natural Forest Resource Protection), due to the management of the timber yield reduction, the use of coal is popularized to compensate for the reduced supply and consumption of firewood. Firewood bioenergy is almost carbon neutral, but the use of coal leads to additional carbon emissions and results in carbon leakage. The off-site GHG emissions generated by the substitution of coal for bioenergy were derived from Eq. (74).

(74) FGC*_t_* = QC*_t_* × EF_c_ × 10^-3^

where FGC*_t_* is the carbon emissions from the substitution of coal for bioenergy (Gg C); QC*_t_* is the additional quantity of coal consumed to compensate for the reduction in firewood supply in the *t^th^* year (t) that is calculated using formula Eq. (75); and EFc is the carbon emission factor of coal, i.e., 0.47 t C/t coal [21].

(75) QC*_t_* = WYF*_t_* / 2

where WYF*_t_* is the reduced firewood yield in the *t^th^* year (m^3^), and 1 ton of coal can substitute for 2 m^3^ of firewood [66].

#### Off-site GHG emissions from the ecological migration (FGE)

We assume that ecological migration was implemented with units of individual houses and that the carbon leakage generated from the transport of these houses was calculated using Eq. (76), (77).

(76) QDE*_t_* = 2 × UDT × DD × RE × WL × NE*_t_* × 10^-6^

where QDE*_t_* is the mass of the diesel fuel consumed in the process of transporting houses in the *t^th^* year (t); UDT is the diesel fuel consumed per truck per hundred kilometers, which is 7 L·t^−1^·100 km^−1^ [26], DD is the diesel density (850 kg·m^−3^); RE is the average distance of relocation, which is 300 km according to ecological migration practices in China [67]; WL is the weight of the load per truck, which we set as 2 tons; and NE*_t_* is the number of households relocated in the *t^th^* year [11], with a factor of 2 is applied for the round trip. Thus, the corresponding off-site GHG emissions from diesel fuel combustion during transport were calculated using the following formula Eq. (77).

(77) FGET*_t_* = QDE*_t_* × EF_D_ × 10^-3^

The off-site GHG emissions from housing construction during ecological migration were derived via the following equation Eq. (78).

(78) FGEH*_t_* = EF_H_ × NH_0_ × NE*_t_* × UH_0_ × 10^-6^

where EF_H_ is the carbon emissions due to constructing a unit area of residential housing, 94.91 kg C·m^-2^ [68]; NH_0_ is the average number of residents per house in rural China, i.e., 4 capita·house^-1^ [44]; and UH_0_ is the average area of housing per person in rural China, i.e., 30 m^2^·capita^-1^ [44].
