# Supplementary material for: Methodology for accounting the net mitigation of China's ecological restoration projects (CANM-EP)
Source: MethodsX. 2019 Jul 19;6:1753–73. doi: 10.1016/j.mex.2019.07.015 (PMC6687229; doi:10.1016/j.mex.2019.07.015)
Supplement: Supplementary file 1 [file mmc1.docx]

**Appendix A1**

**Table A1 Quantity of materials consumed per unit area of each activity in the respective regions of China (kg·ha^-1^)**

| Material | Activity | Northwest | Southwest | Northeast | North | Central south and east | References |
| --- | --- | --- | --- | --- | --- | --- | --- |
| Diesel | Site preparation | 18.00 | 18.00 | 18.00 | 18.00 | 18.00 | [1-2] |
|  | Transportation of seedlings | 3.13 | 3.13 | 4.69 | 3.13 | 3.13 |  |
| Gasoline | Forest patrol | 2.54 | 2.32 | 2.05 | 2.84 | 2.26 |  |
| Steel | Construction of forest protection billboards | 1.02 | 1.02 | 1.02 | 1.02 | 1.02 |  |
|  | Construction of fencing in forests | 5.10 | 5.10 | 4.94 | 5.10 | 4.95 |  |
|  | Construction of fencing on grasslands | - | - | - | 72.17 | - | [3] |
| Water | Irrigation of ecological forest | 40.00×10^3^ | 40.00×10^3^ | 40.00×10^3^ | 40.00×10^3^ | 40.00×10^3^ | [1-2] |
|  | Irrigation of economic forest on barren mountain and land | 265.3×10^3^ | 307.5×10^3^ | 237.0×10^3^ | 159.3×10^3^ | 177.5×10^3^ | [1-2] |
|  | Irrigation of economic forest on converted farmland | 1.25×10^6^ | 0.73×10^6^ | 1.38×10^6^ | 1.05×10^6^ | 0.71×10^6^ | [1-2] |
|  | Irrigation of grass planting | - | - | - | 13.68×10^6^ | - | [3] |
| Cement | Construction of forest road | 892.8 | 892.8 | 892.8 | 892.8 | 892.8 | [1-2] |
|  | Construction of fencing in forests | 3.92 | 3.92 | 3.79 | 3.92 | 3.80 | [1-2] |
|  | Construction of fencing on grasslands | - | - | - | 55.41 | - | [3] |
|  | Construction of feeding greenhouse | - | - | - | 126.6×10^3^ | - | [3] |
| Pesticide | Control of diseases and insects in forests on barren mountain and land | 0.08 | 0.04 | 0.02 | 0.05 | 0.09 | [1-2] |
|  | Control of diseases and insects in forests on converted farmland | 0.09 | 0.05 | 0.03 | 0.06 | 0.11 | [1-2] |
| Herbicide | Weed control on afforestation land | 1.67 | 1.67 | 1.67 | 1.67 | 1.67 | [1-2] |
|  | Tending of young forests on barren mountain and land | 5.21 | 5.05 | 11.68 | 7.86 | 5.19 | [1-2] |
|  | Tending of young forests on converted farmland | 5.35 | 5.35 | 12.04 | 8.02 | 5.40 | [1-2] |
| Fertilizer | Fertilization of economic forests on barren mountain and land | 1114 | 1144 | 1203 | 842.18 | 1201 | [1-2] |
|  | Fertilization of economic forests on converted farmland | 4934 | 4545 | 4374 | 4714 | 4384 | [1-2] |
|  | Fertilization of grass planting | - | - | - | 561.96 | - | [3] |

[1] B.J. Liu, L. Zhang, F. Lu, X.K. Wang, W.W. Liu, H. Zheng, L. Meng, Z.Y. Ouyang

**Greenhouse gas emissions and net carbon sequestration of “Grain for Green” Program in China**

Chinese Journal of Applied Ecology, 27(2016), pp. 1693-1707

[2] B.J. Liu, F. Lu, X.K. Wang, W.W. Liu, L.Y. Wang, E.M. Rao, L. Zhang, H. Zheng

**Greenhouse gas emissions and net carbon sequestration of the Natural Forest Protection Program in China**

Acta Ecologica Sinica, 36(2016), pp. 4266-4278

[3] S.L. Shi

**Technical guidelines for work on grassland**

Jindun Press, Beijing (2009)
